# Human enterovirus 71 protein interaction network prompts antiviral drug repositioning

**DOI:** 10.1038/srep43143

**Published:** 2017-02-21

**Authors:** Lu Han, Kang Li, Chaozhi Jin, Jian Wang, Qingjun Li, Qiling Zhang, Qiyue Cheng, Jing Yang, Xiaochen Bo, Shengqi Wang

**Affiliations:** 1 Department of Biotechnology, Beijing Institute of Radiation Medicine, Beijing 100850, China; 2 Department of Traditional Chinese Medicine and Neuroimmunopharmacology, Beijing Institute of Pharmacology and Toxicology, Beijing 100850, China; 3 State Key Laboratory of Proteomics, Beijing Proteome Research Center, National Center for Protein Sciences (Beijing), Beijing Institute of Radiation Medicine, Beijing 102206, China

## Abstract

As a predominant cause of human hand, foot, and mouth disease, enterovirus 71 (EV71) infection may lead to serious diseases and result in severe consequences that threaten public health and cause widespread panic. Although the systematic identification of physical interactions between viral proteins and host proteins provides initial information for the recognition of the cellular mechanism involved in viral infection and the development of new therapies, EV71-host protein interactions have not been explored. Here, we identified interactions between EV71 proteins and host cellular proteins and confirmed the functional relationships of EV71-interacting proteins (EIPs) with virus proliferation and infection by integrating a human protein interaction network and by functional annotation. We found that most EIPs had known interactions with other viruses. We also predicted ATP6V0C as a broad-spectrum essential host factor and validated its essentiality for EV71 infection *in vitro*. EIPs and their interacting proteins were more likely to be targets of anti-inflammatory and neurological drugs, indicating their potential to serve as host-oriented antiviral targets. Thus, we used a connectivity map to find drugs that inhibited EIP expression. We predicted tanespimycin as a candidate and demonstrated its antiviral efficiency *in vitro*. These findings provide the first systematic identification of EV71-host protein interactions, an analysis of EIP protein characteristics and a demonstration of their value in developing host-oriented antiviral therapies.

Enterovirus 71 (EV71) of the Picornaviridae family is one of the predominant causes of human hand, foot, and mouth disease (HFMD), especially across the Asia-Pacific region^1^. Although HFMD caused by EV71 infection mostly leads to mild symptoms, the disease may be complicated by serious symptoms, such as poliomyelitis-like paralysis, encephalomyelitis, myocarditis and neonatal sepsis, resulting in severe and even fatal consequences in infections of children under 5 years of age^2^,^3^,^4^,^5^. In the absence of efficacious therapy for severe infections and preventive vaccines, outbreaks and recurrent EV71 epidemics are major threats to public health and cause widespread panic during epidemics^1^,^2^,^3^,^4^,^5^,^6^. The EV71 genome is a single-stranded positive RNA that is approximately 7.4 kb in size and contains three encoding regions: P1, P2, and P3. The P1 region encodes the four structural proteins (external VP1, VP2, and VP3 and internalized VP4), whereas the P2 and P3 regions encode the other seven non-structural proteins (2A, 2B, and 2C encoded by the P2 region, and 3A, 3B, 3C, and 3D encoded by the P3 region)^1^. Phylogenetic analysis of EV71 based on the VP1 gene divided this virus into three independent lineages designated A, B and C, of which genotypes B and C still circulate worldwide^7^. Here, we focused on EV71 genotype C, which continues to circulate throughout China.

The rapid progress of systematic host-virus interaction research has provided new data for clinical applications and network-based models for virus infections^8^. To date, little progress has been made in virus infection therapies, such as Tamiflu, which is an influenza A virus self-protein neuraminidase-targeted drug. This limited progress has been attributed to the rapid mutation of viral genomes, which leads to virus-targeted drugs to lose effectiveness against new variants. Concomitant with the rapid progress in systematic attempts to collect host-virus physical interaction data, protein annotation with RNA interference (RNAi) data and other functional data, recently we have witnessed changes in the perspective of antiviral drug research^9^. Drugs targeting essential host factors for viruses may be broad-spectrum and mutant-insensitive. The common method to identify essential host factors for viruses is to knock down host genes and determine whether virus infection is controlled. However, this approach is not practical for every virus due to its low repeat rate and high cost. The virus-host protein-protein interactome also provides potential host targets for antiviral research. The yeast two-hybrid(Y2H) system was introduced twenty-five years ago and has been widely used to investigate protein-protein networks of model organisms and pathogens and hosts, such as bacteria, yeast, fruit flies, tumor viruses and hosts, and herpes viruses and hosts^10^,^11^,^12^. The huge interactome datasets obtained from genome-wide yeast two-hybrid screening have greatly facilitated the identification of crucial proteins that are considered druggable. But the wide distribution of host proteins that interact with viruses makes the selection of drugs targeting multiple host targets difficult. For enterovirus 71, neither genome-wide RNAi screening nor virus-host protein-protein interaction identification has been explored.

Here, we constructed an EV71 protein interaction network, and by integrating public protein and drug databases, we verified the protein functions, explored their potentials in serving as drug targets and predicted anti-viral drugs (Fig. 1). We identified interactions between EV71 proteins and host cellular proteins using a yeast two-hybrid (Y2H) screen to provide a network view of EV71 infection. A viral ORFeome was generated that included ORFs encoding all full-length mature proteins and several protein domains of EV71 BrCr. These bait sequences were cloned into fusion vectors with a GAL4 DNA binding domain. The bait constructs were screened against a human brain cDNA library using a highly stringent Y2H assay. Twenty-nine EV71-human protein interactions were identified. Analysis of the integrated EV71-human protein interaction network revealed the topological features of the EV71-interacting human proteins (EIPs) and the functional pathways related to EV71 infection. Through overlap analysis of EIPs and proteins with interactions or functional associations with other viruses, we found that ATP6V0C might be a broad-spectrum essential host factor and further validated its essentiality for EV71 infection *in vitro*. We then mapped EIPs to known drug targets; the related drug indications mainly covered EV71 infection-related symptoms, indicating the potential of EIPs to serve as host-directed antiviral targets. We used connectivity map resources to find drugs that inhibited EIPs and to help validate the value of EIPs as antiviral targets. The drug tanespimycin showed the most significant inhibition of EIP expression and significantly inhibited EV71 infection *in vitro*.

## Results

### Identification and annotation of EV71-human protein interactions

**Figure 2:**
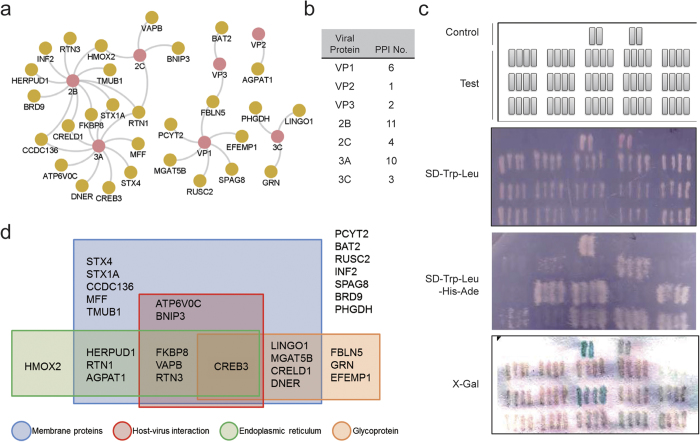
Overview of validated EV71-human protein-protein interactions. (**a**) Validated interactions between EV71 proteins and human proteins; (**b**) Number of human proteins that interacted with each EV71 protein; (**c**) A representative result of a yeast re-transformation assay; (**d**) Venn Diagram for 29 EIPs to show the most common annotations of these proteins.

A comprehensive interactome map between EV71 proteins including VP1, VP2, VP3, VP4, 2A, 2B, 2C, 3A, 3B, 3C and cellular proteins was generated by Y2H screens. A total of 66 positive colonies were obtained. The colony identities were determined by DNA sequencing. Fifty-eight of the colonies were tested with the yeast re-transformation assay. Finally, 42 positive colonies were obtained, which represented 37 unique protein-protein interactions between EV71 and 29 human proteins. We provide a network view of the protein-protein interactions in Fig. 2a. No virus-host protein interactions were identified for the internalized capsid protein VP4 and two other non-structural EV71 proteins (2A and 3B). The non-structural protein 2B and 3A interacted with more host proteins (Fig. 2b and c) and shared as many as five human protein interactions. RTN1 was the only protein with interactions with three viral proteins.

We used the DAVID online tools^13^,^14^ to annotate and cluster the EIPs and to identify common protein characteristics (Supplementary Table S1). Most of the genes encoded membrane proteins, some of which functioned in nerve signal transduction pathways. The four most enriched annotation terms were membrane proteins, endoplasmic reticulum, host-virus interaction and glycoprotein. Only two proteins (STX1A and STX4) were involved in the same KEGG pathway (vesicular transport). Moreover, eighteen of these EIPs were membrane proteins, six were host-virus interaction proteins, and eight were endoplasmic reticulum proteins, including seven proteins located at the endoplasmic reticulum membrane (Fig. 2d, Supplementary Table S1).

### Construction of the EV71-human protein interaction network and topological analysis

**Figure 3:**
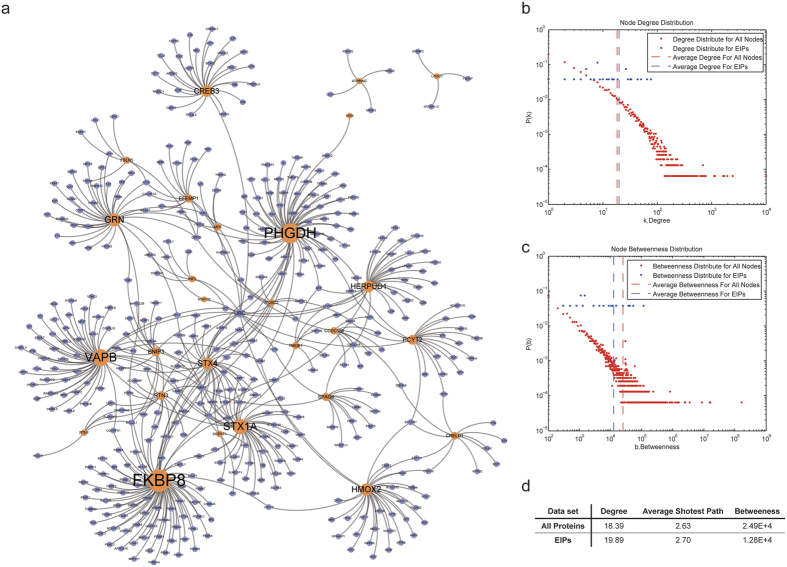
The EAP interaction network and the topological analysis of EIPs in the human protein interactome. (**a**) The protein interaction subnetworks between EAPs, with orange nodes representing EIPs and blue nodes representing the human proteins that interact with them. The node sizes are directly proportional to the node’s degrees in this subnetwork; (**b**,**c**) The degree (**b**) and betweenness centrality (**c**) distributions of all proteins (red points) and EIPs (blue points) in the human protein-protein interaction network. *P*(k) represents the probability of a node having a degree of k, *P*(b) represents the probability of a node having a betweenness centrality of (**b**). The dashed lines represents the average degrees of all proteins (red) and EIPs (blue); (**d**) The average degree, betweenness centrality and shortest path for the EIPs and all proteins.

Using the Biological General Repository for Interaction Datasets (BioGRID) Database^15^ as a reference, we constructed a human protein-protein interaction (PPI) network containing 141,792 non-redundant physical PPIs between 15,422 human proteins. As many as twenty-seven (93.1%) EIPs were found to interact with other human proteins. These proteins together with another 424 proteins formed a protein interaction network with 527 non-redundant physical interactions (Fig. 3a), including 2 interactions between EIPs (i.e., between VAPB and STX4 and between VAPB and STX1A) and 7 EIP self-protein interactions (BNIP3, CREB3, FBLN5, FKBP8, MFF, RTN3 and STX1A). We defined the EIPs together with the other 424 EIP-interacting proteins as EV71 infection-associated human proteins (EAPs).

To investigate the centrality of EIPs in a human PPI network (i.e., to determine whether these proteins tended to mostly interact or to act as bridges between other proteins), we calculated the degree, betweenness centrality, and average shortest path length of each protein in the human PPI network, including the EIPs (Fig. 3b,c and d). The degree equals the number of proteins with direct interactions with a given protein, which represents local centrality. The shortest path between two proteins is the shortest route that contains the least number of proteins and the interactions between them in the network. The betweenness centrality counts the ratio of the shortest paths between two proteins in the network that passes through given proteins, representing a global centrality. The average degree, average shortest path, and average betweenness centrality of all proteins in the human PPI network were 18.39, 2.63 and 2.49 × 10^4^, respectively, whereas these values for the EIPs were 19.89, 2.70 and 1.28 × 10^4^, respectively (Fig. 3b,c and d). The significance of the differences between the average degrees and average betweenness centralities of the EIPs and all proteins were tested using the two-sided Wilcoxon rank sum test at the 1% significance level (*p* = 3.53 × 10^−3^ and 5.83 × 10^−3^, respectively), although the average shortest path lengths were not significantly different. The higher degree indicated that the EIPs were the proteins with the most interactions, whereas the lower betweenness centrality indicated that the EIPs were not central in the global human interactome. Considering the small number of identified EIPs and the slight differences, the credibility of the conclusions needed further verification.

### Functional analysis of the EV71-human protein network

To explore the EIP functions, we analyzed the EIP enrichment significance in different pathways. The Reactome pathway database is an open-source and peer-reviewed pathway database that provides pathway knowledge^16^,^17^. We analyzed pathway enrichment for EIPs (Supplementary Table S2). Unexpectedly, the pathways in which the EIPs were enriched were decentralized, with no more than two proteins found in the same pathway. Using *p* < 0.01 as the cutoff, nine Reactome pathways were identified as significantly enriched, including neuronal system pathways, such as botulinum neurotoxicity and SNARE complex-related pathways, metabolic pathways, such as serine biosynthesis and glycerophospholipid biosynthesis, and extracellular matrix organization pathways, such as elastic fiber formation (Supplementary Table S2).

Due to the small number of EIPs, the functional annotation and enrichment analysis provided limited information. To obtain more detailed function annotations, the EAPs were used to analyze the enrichment significance in the Reactome pathways (Supplementary Table S3). Nine of the twenty-four top level Reactome pathways (Apoptosis, Cell Cycle, Disease, DNA Replication, Gene Expression, Membrane Trafficking, Metabolism, Immune System and Neuronal System) covered the 160 significantly enriched Reactome pathways (*p* < 0.01) (Supplementary Table S3), suggesting that the EAPs functioned in various bioprocesses. Two of the most significantly enriched pathways (botulinum neurotoxicity and S phase) are shown in Figure S1.

Due to the different functions of the EV71 proteins, the host proteins interacted with different viral proteins and possibly different bioprocesses or pathways. We divided the EAPs into four groups according to their interacting viral proteins to analyze their enriched Reactome pathways (Supplementary Tables S4–S8). For the EAPs associated with the capsid proteins (VP1, VP2 and VP3), the enriched pathways were mainly the TGF-beta-SMAD signal pathways (Supplementary Table S8). By contrast, the viral 2B protein-associated EAPs were involved in more widely distributed pathways, including the cell cycle G1, S and M phase-related pathways, immune system-related pathways, such as activation of NF-kappa B in B cells, class I MHC-mediated antigen processing and presentation and inflammasomes, signal transduction pathways, such as beta-catenin-independent WNT signaling, mTOR signaling and signaling by ERBB2, neuronal system pathways, such as neurotransmitter release cycle and botulinum neurotoxicity, and other pathways, such as HIV infection, ubiquitin-dependent degradation and apoptosis regulation (Supplementary Table S4). Most of these pathways also closely interacted with viral protein 3A. Additionally, the 3A-associated EAPs were enriched in DARPP-32 events, transmembrane transport of small molecules and lysosome vesicle biogenesis pathways. Viral protein 2C-associated EAPs were involved in relatively fewer types of pathways, which primarily included neuronal system pathways, such as botulinum neurotoxicity and acetylcholine neurotransmitter release cycle, and metabolic pathways, such as metabolism of lipids and lipoproteins (Supplementary Table S6). The viral protein 3C-interacting proteins and their neighbors were primarily enriched in the cellular response to stress pathways, such as the cellular response to hypoxia and cellular senescence (Supplementary Table S7).

### Overlap analysis of EIPs and other virus-interacting proteins identified ATP6V0C as a potential broad-spectrum antiviral target

With the rapid growth of virus-host protein interaction knowledge, host-virus interactions can be regarded from a network perspective. The large number of systematically validated virus-host interactions can serve as a baseline to estimate the values of each EIP during virus infections. The most common experimental methods for the systematic identification of virus-interacting host factors have primarily identified viral proteins that physically interact with host proteins (virus-targeted proteins, VTPs) using Y2H screens^18^ or essential host factors (EHFs) using genome-wide RNAi screening^19^,^20^. We systematically collected virus-protein physical interactions and RNAi screening results to determine whether the EIPs were also functional for other virus infections. Twelve out of 29 EIPs were VTPs for 11 other viruses (Supplementary Table S9), and 11 out of 29 EIPs were EHFs for 8 other viruses (Supplementary Table S10).

**Figure 4:**
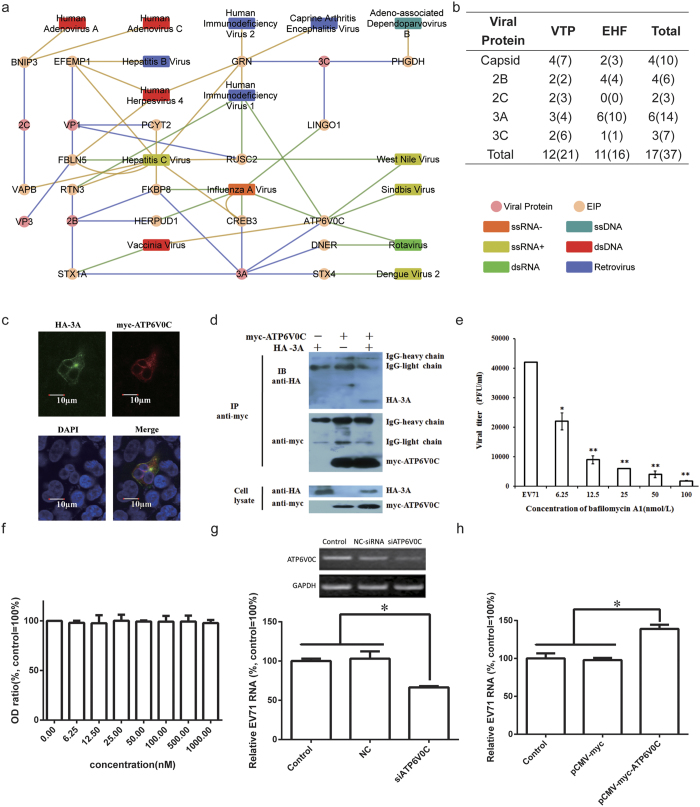
Overlap analysis of EIPs and other virus-interacting proteins. (**a**) Blue edges represent physical interactions between the viral proteins and EIPs, and orange and green edges represent EIP VTP and EHF interactions to the corresponding viruses; (**b**) The statistical analysis of other virus-interacting EIP numbers with the corresponding interaction numbers marked in parentheses; (**c**) Co-localization of ATP6V0C and 3A in the co-transfected RD cells; (**d**) The interaction between ATP6V0C and 3A was confirmed by co-immunoprecipitation; (**e**) Inhibition of EV71 replication by bafilomycin A1. RD cells were infected with the EV71 virus in the presence of varying concentrations of bafilomycin A1 (0, 6.25, 12.5, 25, 50 and 100 nmol/L) for 1 h. After washing, the cells were cultured in fresh growth mediumfor an additional 12 h. The cell cultures were subjected to plaque assays. Compared with virus control, *P < 0.05, **P < 0.01, ±s, n = 3; (**f**) Cytotoxicity of bafilomycin A1. Percent viability of RD cells was determined normalized to the absorbance at 450 nm of a drug-free culture; (**g**) Inhibition of EV71 propagation by ATP6V0C siRNA; (**h**) Increased EV71 propagation by ATP6V0C. RD cells were transfected with ATP6V0C siRNA or pCMV-myc-ATP6V0C and subsequently infected with EV71. 24 h later, the virus RNAs in cell cultures were isolated and subjected to real time RT-PCR. Virus growth is shown as the average percentage relative to the control cells infected with EV71. *P < 0.05, ±s, n = 3.

We present the EIPs and their relationships to other viruses from a network perspective in Fig. 4a. EIPs with known physical interactions with other viruses or that served as EHFs for other viruses were sorted into five categories according to their interacting EV71 proteins (Fig. 4b). The capsid protein-interacting host proteins were more likely to be known VTPs. Half of the capsid-interacting proteins were known VTPs, indicating that these proteins were universal proteins for viral adhesion. Interestingly, 6 out of 11 3A-interacting proteins were EHFs, indicating the increased functional importance of the 3A-interacting host proteins.

For the VTPs, Granulins encoded by the GRN gene was reported to interact with as many as five viruses, and EFEMP1 was reported to interact with three viruses. Due to the relatively few genome-wide RNAi screening reports, most EIPs were reported to be EHFs of only one virus. However, RUSC2 and ATP6V0C were EHFs for two and five different viruses, respectively. The broad relationships between ATP6V0C and different viruses indicated its universally important functions during different virus infections, making it a potential broad-spectrum host target for antiviral therapies. Therefore, we validated the interaction between ATP6V0C and the EV71 3A protein and studied the function of ATP6V0C in EV71 infection. Results of the subcellular co-localization ATP6V0C and 3A in co-transfected RD cells showed that ATP6V0C and 3A co-localized primarily in the cytoplasm (Fig. 4c). Additionally, results of co-immunoprecipitation experiments indicated that ATP6V0C interacted with the 3A protein in co-transfected RD cells(Fig. 4d). To study the role of ATP6V0C in EV71 infection, the ATP6V0C siRNA were used to downregulate the ATP6V0C gene expression and the pCMV-myc-ATP6V0C which could express ATP6V0C protein in cells were used to overexpress ATP6V0C. Results showed that siRNA targeting ATP6V0C inhibited the EV71 propagation (Fig. 4g),and that overexpressing ATP6V0C in cells enhanced the EV71 propagation(Fig. 4h).

Bafilomycin A1 is a selective inhibitor of vacuolar H + ATPases (V-ATPases). In this study, bafilomycin A1 was used to further study the function of APT6V0C in EV71 infection. RD cells were infected with the EV71 virus in the presence of varying concentrations of bafilomycin A1 (0, 6.25, 12.5, 25, 50 and 100 nmol/L) for 1 h. After washing three times with PBS, the cells were cultured in fresh growth medium for an additional 12 h. The cell cultures were collected and subjected to plaque assays. The results showed that bafilomycin A1 inhibited EV71 infection in a dose-dependent manner (Fig. 4e). Additionally, the *in vitro* cytotoxicity results showed that treatment of RD cells with serial bafilomycin A1 dilutions for 1 h was not cytotoxic (determined by the cellular proliferation rates) even at a concentration as high as 1000 nM (Fig. 4f).

### Drug prediction with EIPs

The high overlap rate of EIPs with other virus-interacting proteins proved the reliability of EIPs and the possibility of their use as broad-spectrum host-dependent antiviral targets. However, whether the drugs targeting them could be effective was unknown. To explore the potential use of these proteins as host targets, we queried DrugBank to identify drugs targeting them. Contrary to the many overlaps between the EIPs and VTPs or EHFs, few EIPs were known drug targets. Only 2 out of the 29 EIPs were targeted by only 2 drugs: NADH targeting PHGDH and HMOX2 and Stannsoporfin targeting HMOX2. NADH is an endogenous small molecule that participates in the citric acid cycle and cellular respiration. Too many proteins were recorded as targets of NADH, making PHGDH and HMOX2 non-specific. The endogenous character and non-specific targets made NADH an unsuitable drug candidate. Moreover, Stannsoporfin failed to inhibit EV71 infection *in vitro*. To obtain more drug candidates, we listed all drugs targeting EAPs in Supplementary Table S11. The drug candidates increased to 567 when the EIP neighbors were considered. These drugs had 743 interactions with 107 different targets; the interaction type and drug properties, such as the drug type, category, and Anatomical Therapeutic Chemical (ATC) code, are also listed in Supplementary Table S11. An enrichment analysis of drug properties could help determine the drug characteristic tendencies and EAP functional features. We listed the most enriched (*P* < 10^−10^) drug properties in Table 1 (detailed in Supplementary Table S12). The enriched drug types were diverse and included approved, psychotropic and neurological drugs, beta-blocking agents, glucocorticoids and anti-inflammatory agents. These drugs were primarily related to inflammation control and neurological indications, which were related to EV71 infection symptoms. The drug-target interaction types were also counted and analyzed. The inhibitor, agonist and negative modulator types were the most enriched (*P* < 10^−3^) drug-target interaction types (Supplementary Table S13).

Although the target-associated strategy provided sufficient host-oriented anti-EV71 drug candidates, these candidates usually only targeted one or two proteins, and few of them directly targeted EIPs. Thus, one by one validation of their antiviral activities would be difficult. Ideally, we would like to identify a drug that inhibits all EIPs to block host-virus interactions. The connectivity map^21^,^22^ and Gene Set Enrichment Analysis (GSEA)^23^ provided an applicable strategy for the evaluation of drug influences on a group of targets (i.e., signatures through expression profiles). The 6,100 pairs of expression profiles representing the cellular responses to 1,309 different drugs were arranged in 6,100 gene rank lists in connectivity map 2.0^22^. These genes were ranked by expression changes due to drug interference (i.e., the genes up-regulated by drug interference were placed on top of the rank list and the down-regulated genes were placed on the bottom). GSEA based on the Kolmogorov–Smirnov (K-S) statistic was used to estimate whether a group of targets (i.e., a signature) tended to appear at the top (or bottom) of the gene rank lists to determine whether the signature was universally up-(or down-) regulated by drug interference. The enrichment scores acquired by GSEA were in the range of −1 to 1, with negative scores indicating inhibition of the signatures and positive scores indicating activation of the signatures. Here, we used EIPs as the signature and attempted to find drugs that inhibited them. All 6,100 drug interference experiments were sorted by their corresponding enrichment scores to EIPs in ascending order (i.e., the top-ranked drug interference experiments tended to inhibit EIPs). Experiments using the same drug were checked to determine whether they were consistently top-ranked. The permutation *p* values estimated with 1,000,000 random rankings of the 6,100 experiments as a baseline were used to describe the significance of a drug’s inhibition of EIPs. Using a permutation *p* < 0.01 as the cut off, 27 of the 1,309 drugs were identified to significantly inhibit EIP expression (Supplementary Table S14).

**Figure 5:**
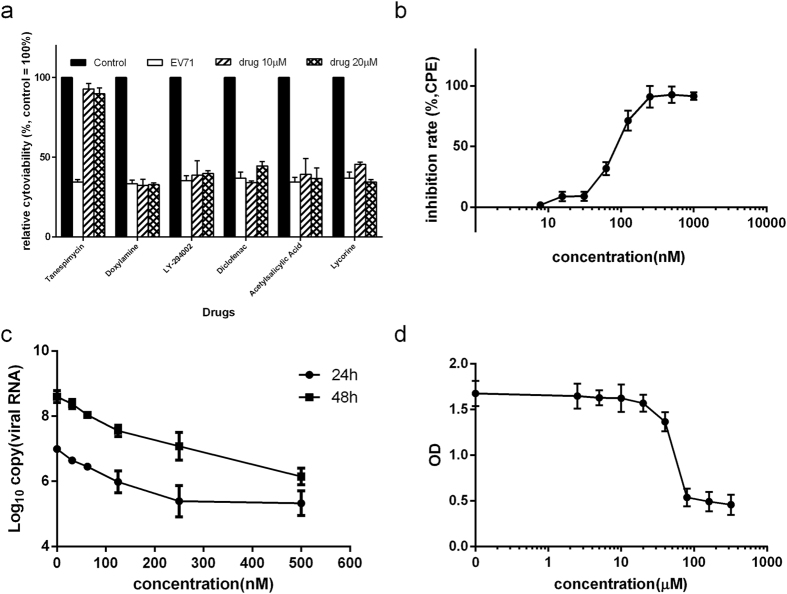
The anti-EV71 activity of drugs identified as significantly inhibiting EIPs. (**a**) Relative viability of EV71-infected cells in response to eleven drugs. RD cells were seeded into 96-well plates and infected with EV71 at an MOI of 1 for 1 hr. Then, the cells were treated with small-molecule drugs at final concentrations of 10 μM and 20 μM. Cell viability was detected using the CCK8 assay at 48 hr post-infection, and signals from the mock-treated reaction were set as 100%; (**b**) The anti-EV71 activity of tanespimycin in the CPE assay. RD cells were seeded into 96-well plates and infected with EV71 at an MOI of 1 for 1 hr. Then, the cells were treated with various doses of tanespimycin. Tanespimycin-mediated cell viability was detected using the CCK8 assay at 48 hr post-infection; (**c**) The anti-EV71 activity of tanespimycin based on the detection of viral RNA using real-time RT-PCR. RD cells were seeded into 96-well plates and infected with EV71 at an MOI of 1 for 1 hr. Then, the cells were treated with various doses of tanespimycin. After incubation for 24 h and 48 h, the cell culture supernatants were harvested. Viral RNA was isolated and subjected to real-time RT-PCR; (**d**) Tanespimycin cytotoxicity. RD cells were treated with various tanespimycin concentrations for 48 h. Cell viability was tested using a Cell Counting Kit-8 assay. All tests were performed independently three times. The results are expressed as the mean ± SD of three samples.

The top-ranked drug tanespimycin was chosen and evaluated to detect antiviral activity against EV71. The results are shown in Fig. 5a. Tanespimycin significantly inhibited virus-induced cytopathic effects (CPEs) at the 10 and 20 μM concentrations. To validate the anti-EV71 activity of tanespimycin, the CC_50_ and EC_50_ of tanespimycin against EV71 were calculated (Fig. 5b,c and d). Figure 5d shows the cytoxicity of tanespimycin in RD cells. The CC_50_ was calculated as 102.50 μM. To assess the antiviral activity, the RD cells were infected with EV71 for 1 hour, followed by the addition of serial dilutions of tanespimycin. After 48 hours, virus-induced CPE was detected using the CCK-8 assay. The cell cultures were collected and viral RNA was isolated to detect EV71 RNA using real-time RT-PCR. The results are shown in Fig. 5b and c. Tanespimycin inhibited the virus-induced CPE and viral RNA level in a dose-dependent manner. The EC_50_ of CPE inhibition was 85.96 nM.

## Discussion

A protein interaction network was generated from a collection of host proteins that interacted with EV71 viral proteins to identify potential associations between virus proliferation, virus-induced cytotoxicity, host defense escape and host-virus interactions and to find potential therapies by blocking host-virus interactions.

The functional and association analysis of EIPs provided a hypothesis for infection-dependent pathways and potential therapies. However, whether these interactions have functional influences requires validation. These results provided an initial starting point for a complex systems analysis of EV71 infection. Obtaining a systematic understanding of virus infection depends on the confirmation of each component and the integration of the information from the entire cellular function network. Some of our results agreed with known knowledge of EV71 infection. For instance, STX1A and STX4 have roles in the same KEGG pathway (vesicular transport). Their interactions with the 2B and 3A proteins agreed with the finding that 2B and 3A promoted vesicular transport during virus infection^24^.

Some viral proteins interacted with human proteins with high local and global centralities^25^,^26^. The high local or global centralities of human proteins indicated that these proteins tended to interact with more proteins or have larger influences on signal transfer throughout the human protein interaction network, respectively. The literature analysis indicated that the tendency for viral proteins to interact with central proteins might be a general hallmark of viral proteins^26^. The low global centralities indicated relatively weak influences of EIPs on the whole human protein interaction network, which might result from the limitations of the tissue-specific protein library used in this study. The functional analysis of EIPs and their neighboring proteins revealed that their major functions were most likely perturbed by viral proteins during infection. These functions included the cell cycle, apoptosis, multiple signaling transfer pathways, immune system and neuronal system pathways. Some of these pathways, such as the cell cycle and apoptosis-related pathways, were closely related to virus proliferation and transmission, and some of these pathways, such as botulinum neurotoxicity, inflammasomes and other immune system pathways, might play important roles in acute infection symptoms. These results provide insights for further investigations into host-virus interactions and suggest that the proteins that directly interacted with viruses could be essential for virus replication or disease progression and thus serve as potential antiviral targets.

Before investigating the potential of EIPs for drug repositioning, we explored the associations between EIPs and other viruses. The results suggested that more than half of the EIPs had known interactions with other viruses, indicating their potential to serve as broad-spectrum targets. The most outstanding EIP (i.e., ATP6V0C) was also an EHF for five different viruses. The *in vitro* validation of the influence of EV71 infection in this report demonstrated its essentiality. ATP6V0C is subunit c of the vATPase. Reports have shown that ATP6V0C or vATPase is essential for the entry of many viruses, including influenza virus^27^, human cytomegalovirus^28^, West Nile virus^29^, polio virus^30^, and Sindbis virus^31^. According to the role of vATPases in other virus infection and our results, we speculated that the possible mechanism by which ATP6V0C facilitated EV71 infection might be via its effect on the entry of EV71 into cells.

The association of EIPs with drugs could help elucidate the host-oriented antiviral potential of known drugs. The approved drugs were significantly outnumbered in the enrichment analysis of target-driven drug candidates, suggesting safe and convenient drug repositioning, although the significance may result from knowledge bias. The psychotropic and neurological drugs were enriched due to the outnumbered drug targetable neural proteins in the EAPs. Other drug properties, such as beta-blocking agents, glucocorticoids and anti-inflammatory agents, indicated possible adjuvant drugs for EV71 infection. Despite massed indirect target-driven drug candidates, few safe drugs directly target EIPs. Because drugs affect few to a large number of targets, specificities of target-driven drug candidates were different. Drugs with many targets are more likely to be elected as candidates. The EIPs PHGDH and HMOX2 are targets of NADH. While considering the fact that NADH affects hundreds of targets and dozens of pathways, it is probably a false positive prediction. We attempted to use a connectivity map, which is another powerful strategy for drug repositioning^21^,^22^, to predict drug candidates with the principle that drug candidate should tend to inhibit EIP gene expression. The *in vitro* validation of tanespimycin’s inhibition of the CPE of EV71 supported this strategy. While the other five high-ranking drugs failed to reduce EV71-induced CPE. As the fuctional importance of each EIP is different, the mere assessment of the overall expression levels of EIPs is practical but loose. A better approach is to identify the functional roles of each EIP, and to find drugs that exactly modulate key EIPs, making it more likely to find effective drugs.

A prominent advantage of host-targeted antiviral strategies is that the host cells provide a much broader target drug candidate than the virus. Moreover, due to the mutation rate of human genes is far lower than the viral gene, the host-targeting strategy can also better solve the problem of drug resistance^32^. In addition, the same host protein may be pivotal for a variety of viruses, therefore, host-targeted drugs may have broad-spectrum antiviral activity^33^. Despite the benefits of host-targeted antiviral strategies, current therapeutic drugs against viral diseases mainly targets the viral components, especially viral enzymes^34^. As viral proteins interact with host factors to promote viral replication and disease process, systematically experimental identification of virus-interacting host factors and bioinformatics methods were used for building virus-host interactomes and predicting therapeutic targets^29^,^35^,^36^,^37^. Although these approaches are still in infancy, more and more antiviral molecules with reduced safety risk and low resistance induction are predicted and validated^34^,^38^,^39^. In this study, public databases were used to functionally annotate EIPs and find EIPs with therapeutic values, and two drug repositioning strategies were used to predict antiviral drugs. Though imperfect, this study provides a pipeline from virus-host interaction network to therapeutic applications. With the accumulation and sharing of virus-host interactome data and progress of systems biology, more host-targeted antiviral drugs will arise.

## Material and Methods

### Reverse transcription PCR (RT-PCR)

Total RNA from EV71 was reverse transcribed into cDNA using a SuperScript^®^ III First-Strand Synthesis System (Invitrogen,California,USA) according to the manufacturer’s instructions. To clone the EV71 PCR amplification gene products in frame with *GAL4DB*, the cDNAs were subjected to PCR amplification using 10 pairs of primers with adapters containing restriction sites in the appropriate positions.

### Construction of the DB fusion plasmid

After digestion with the corresponding restriction enzymes, the DNA fragment and pDBLeu plasmid were added to the ligation reaction mixture. The ligation reactions were incubated overnight at 16 °C and used directly for bacterial transformation. To identify and confirm the ORF entry clones, PCR was performed, followed by sequencing of the plasmids.

### Yeast two-hybrid (Y2H) screens

The bait plasmids were transformed into yeast strain AH109 (Matchmake yeast transformation system 2, Clontech). To test the autoactivation of the bait, the yeast were selected on minimal medium. Then, bait constructs without self-activation were screened against an adult brain cDNA library. Positive colonies that grew on the selection plates were picked. The prey plasmids were isolated and transformed into *E. coli*. The plasmids were re-tested by transformation with the viral bait into yeast AH109. The identities of the positive colonies were identified by DNA sequencing.

### Construction of the EV71 protein interaction network

We downloaded the BioGRID^15^ Protein-Protein Interaction databases from http://thebiogrid.org/versioned 3.2.117 compiled on September 25, 2014. Physical interactions between human proteins were filtered to serve as the human PPI baseline. We mapped the EV71-interacting proteins to the HPRD protein-protein interaction network to obtain physical interactions between these proteins and their neighbors in the human PPI network.

### Topological analysis of EIPs in a human PPI network

We used the Complex Networks Package (Version 1.6) (Self-emergence of knowledge trees: extraction of the Wikipedia hierarchies) to perform the topological analysis. The degree of a protein (node) equals the PPI interactions incident to the node in the whole network. The degree distribution of the EIPs was compared to the degree distribution of whole human proteins. The shortest path is the path between two proteins when the count of the edges in the path is minimized. The shortest path length equals the edge count of the shortest path. Thus, if the number of shortest paths between two different nodes *a* and *b* is expressed as *N*_*ab*_ and the number of shortest paths between *a* and *b* and pass through node *c* is expressed as *N*_*ab*_(*c*), then the betweenness centrality of *c* is expressed as follows:


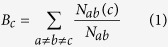


### Annotation clustering with DAVID

The online tool Database for Annotation, Visualization and Integrated Discovery (DAVID) version 6.7^40^ was used to annotate the EIPs. The EIPs were submitted to the DAVID tool and human proteins were used as the baseline for the functional EIP clustering. The default settings were used for all parameters and annotation categories. The annotation clustering results were manually checked to determine the most outstanding characteristics of the EIPs. The EIPs were graphically presented as a Venn diagram according to their main characteristics.

### Reactome pathway enrichment analysis

The Cytoscape plugin “Reactome FI”^16^ version 5.0.0 was used to analyze the pathway enrichment significance and to visualize EAP pathways. The Reactome pathways were loaded into Cytoscape. The EAPs or independent viral protein-interacting proteins were arranged to separate the gene sets. These gene sets were loaded to analyze pathway enrichment. The enrichment *p* value and the false discovery rate for all pathways were provided by the Reactome FI. And pathways with enrichment *p* values less than 0.01 were used.

### Collection of viral physically interacting proteins

The viral physically interacting (targeted) proteins (VTPs) were collected and extracted from open published literature. All VTP data were manually curated and verified by domain experts.

### Collection of essential host factors for virus infection

The essential host factors (EHFs) for virus infection were collected in our previous work^20^. We selected essential host factors for virus infection to form our collection.

### Prediction of candidate drugs with DrugBank

The version 4.0 XML file describing all drug information was downloaded from DrugBank^41^ on November 2, 2014. Drugs with targets that overlapped with EIPs or their neighbors were extracted from the database. Basic information, such as the drug category, ATC code and drug-target action, of these drugs was also collected.

### Enrichment analysis of drug properties

The drug properties of interest included the drug type (small molecule or biotech), drug group (approved information), drug category and ATC code. Briefly, ATC code level 2 was used to represent the drug ATC characteristics. Using all of the drugs in DrugBank as the background, the enrichment significance of drug properties was evaluated by the hypergeometric distribution. Suppose the total drug number in DrugBank is *N* and of the total drug candidate number is *n. K* out of the total drugs are characterized by characteristic x, and *k* out of the drug candidates are characterized by x. The enrichment *p* value of characteristic x is expressed as follows:


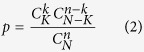


### Enrichment analysis of drug-target actions

Similar to the enrichment analysis of drug properties, the drug-target action types for the drug candidates and total drugs were analyzed. Suppose the number of drug-target actions for total drugs is *N* and that the corresponding number for the drug candidates is *n. K* out of *N* actions are of action type x, and *k* out of *n* actions are of the same type. Then, the action type enrichment significance of the drug candidates is expressed by the enrichment *p* value listed below:


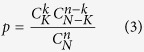


### Prediction of candidate drugs with Connectivity Map

The gene rank list data were downloaded from Connectivity Map version 2.0^22^. The gene rank lists represent gene expression changes in drug stimulation experiments (i.e., the genes at the top (bottom) of the ranked list are the most up- (or down-) regulated genes during the corresponding drug stimulation. The enrichment score for the EIPs in each gene rank list was computed with the Kolmogorov-Smirnov statistic^42^. Supposing the total length of the gene list is *n* and the EIPs corresponded genes are ranked *R*(*i*) where *i* = 1, 2, 3, …, *k*. Then, compute the following values:


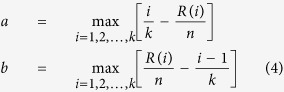


The enrichment scores of the EIPs were set to *a* if *a* > *b* or to −*b* if *b* > *a*. Positive scores indicated EIP activation, whereas negative scores indicated EIP inhibition. The average enrichment score for the same drug was generated from the enrichment scores of each experiment.

To evaluate the significance of a drug’s inhibition of EIPs, the experiments were ranked in ascending order. Supposing there are *n* experiments and *k* out of these experiments are experiments for the same drug. These experiments are ranked *R*(*i*), where *i* = 1, 2, 3, …, *k*. Then, compute the following values:


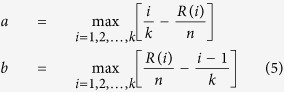


Set *KS* = *a* when *a* > *b* or *KS* = *b* when *a* < *b. N* = 1,000,000 repetitions of random sorted instances used to compute the corresponding *KS*_i_, where i = 1, 2, 3, …, *N*. The permutation *p* value was set to the frequency of *KS*_*i*_ < *KS*.

### Cells, virus and compounds

Rhabdomyosarcoma (RD) cells (American Type Culture Collection, ATCC CCL-136) and Vero cells (ATCC CCL-81), which are frequently used to isolate EV71 from clinical specimens, are highly susceptible to EV71. The cells were routinely grown in Dulbecco’s modified Eagle’s medium high glucose (DMEM, Gibco, Grand Island, NY, USA) supplemented with 10% fetal calf serum (FCS). The EV71 BrCr strain was propagated in the RD cells and titrated in a 50% tissue culture infective dose (TCID50) assay. All compounds were purchased from Sigma-Aldrich (Sigma-Aldrich, St. Louis, MO, USA) and dissolved in water or 10 mM DMSO according to the solubility of the compounds. The final DMSO concentration in the culture medium did not exceed 0.1% and had no antiviral effects. The sense sequence of ATP6V0C siRNA was 5′ CAGCCACAGAAUAUUAUGUAATT 3′^27^ and were synthesed in Shanghai Genepharma Co.,Ltd. The sense sequence of negative control siRNA(NC siRNA) was 5′ UUCUCCGAACGUGUCACGUTT 3′.

### Double-label immunofluorescence assays

The RD cells were cultured on glass cover slips in the bottom of cell culture dishes. At 24 h post-transfection, the cells were fixed for 30 minutes with 4% paraformaldehyde prepared in PBS, permeabilized with 0.1% Triton X-100 in PBS for 15 minutes, washed 3 times with PBS, and incubated at room temperature for 30 min in blocking buffer (2% BSA-PBS). The cells were incubated with the primary antibodies (anti-HA 1:100 and anti-myc 1:100) at 37 °C for 1 h, washed 3 times with PBS at room temperature, and then incubated with the secondary antibodies (anti-rabbit IgG-cy2 1:1000 and anti-mouse IgG-TRITC 1:2000) at 37 °C for 1 h. The cells were counterstained with DAPI and visualized by confocal laser scanning microscopy (Olympus™, Fluoview 300, Japan).

### Immunoprecipitation and western blot analysis

After 24 h, the transfected RD cells were lysed with Tris-HCl buffer (pH 7.4) containing proteinase inhibitor cocktail (Roche, Indianapolis, IN), 150 mM NaCl, 1% NP-40, and 0.25% sodium deoxycholate. The cell lysates were incubated with an anti-myc monoclonal antibody (Cell Signaling Technology) overnight at 4 °C with rotation. The samples were incubated with protein A/G agarose beads (Santa Cruz Biotechnology, Santa Cruz, CA) for 2 h at 4 °C with rotation. After washing 3 times with Tris-HCl buffer, the immunocomplexes captured by the protein A/G agarose beads were boiled for 10 min at 95 °C and resolved by 10% SDS-polyacrylamide gel electrophoresis. The separated proteins were transferred to PVDF membranes (Millipore, Bedford, MA, USA), and the membranes were incubated overnight at 4 °C with the anti-HA or anti-myc antibody. After washing, the membranes were incubated with a horseradish peroxidase-conjugated secondary antibody for 1 h at room temperature. Finally, the membranes were incubated with enhanced chemiluminescent (ECL) reagents (Millipore,) and exposed to autoradiography film in the dark.

### Compound treatments and infection of cells in tissue culture

RD cells (8–10 × 10^3^) were seeded into a 96-well plate. Twenty-four hours later, the cells were infected with EV71 at a multiplicity of infection (MOI) of 1 for 1 h. Then, the medium was replaced with a maintenance medium containing 2% FCS and a serial dilution of compounds. Forty-eight hours later, the cells were observed for virus-induced cytopathic effects (CPEs), and the supernatants were collected for the detection of EV71 RNA or plaque assays.

### Cell proliferation and viability assay

Pathological changes in cells infected with EV71 were observed using an inverted microscope (Olympus CKX41). Cell viability was tested using a Cell Counting Kit-8 (CCK-8, Dojindo Laboratories, Kumamoto, Japan). CPE inhibition data were calculated according to the absorbance measured at 450 nm with a microplate reader (Model 680, Bio-Rad, Hercules, CA, USA) and expressed as the 50% effective (viral CPE inhibitory) concentration (EC_50_). Each experiment was performed in triplicate and repeated three times.

### Plaque assays

Monolayers of Vero cells (3 × 10^5^ per well) were seeded into 12-well plates. After 14 h in culture, the cells were inoculated with diluted viral suspensions for 1 h. The cells were washed with PBS and overlaid with warm 1% methyl cellulose (MC, Sigma, USA) in MEM. After incubation for 4 days, the cells were fixed with 4% formaldehyde for 1 h. The MC was washed and subsequently stained with 1% crystal violet. The virus titer was expressed as the plaque-forming units (PFU) per ml.

### Real-time reverse transcription (RT) PCR to detect the EV71 RNA

Viral RNA was isolated from the cell supernatant using an RNeasy Mini Kit (QIAGEN, Hilden, Germany). Real-time RT-PCR was performed using a Quant One Step qRT-PCR (Probe) Kit (TIANGEN) with EV71-specific primers and probes as previously reported^43^. The forward (fp) and reverse (rp) primers were EV71-1F (5′-CAAGTCTCAGTGCCATTTAT-3′, nt 3002–3021) and EV71-1R (5′-ATTCGGGCATGCCCCATACT-3′, nt 3118–3099), respectively, and the probe was 5′-TCACCTGCAAGCGCATACCAATGGT-3′ (nt 3023–3047). The absolute quantification of the viral RNA was performed using a standard curve.

### *In vitro* cytotoxicity assays

The cytotoxicity of compounds for RD cells was evaluated as the 50% cytotoxic concentration (CC_50_ in μM). Briefly, monolayer cultures of RD cells were exposed to several concentrations of compounds in maintenance medium (2% FCS) at 37 °C for 2 days. Cell viability was assayed using the CCK8 method following the manufacturer’s instructions. Each experiment was performed in triplicate and repeated three times.

## Additional Information

**How to cite this article**: Han, L. *et al*. Human enterovirus 71 protein interaction network prompts antiviral drug repositioning. *Sci. Rep.* **7**, 43143; doi: 10.1038/srep43143 (2017).

**Publisher's note:** Springer Nature remains neutral with regard to jurisdictional claims in published maps and institutional affiliations.

## Supplementary Material

Supplementary Information

Supplementary Table S3

Supplementary Table S4

Supplementary Table S5

Supplementary Table S6

Supplementary Table S7

Supplementary Table S8

Supplementary Table S9

Supplementary Table S10

Supplementary Table S11

Supplementary Table S12

Supplementary Table S13

Supplementary Table S14

## Figures and Tables

**Figure 1:**
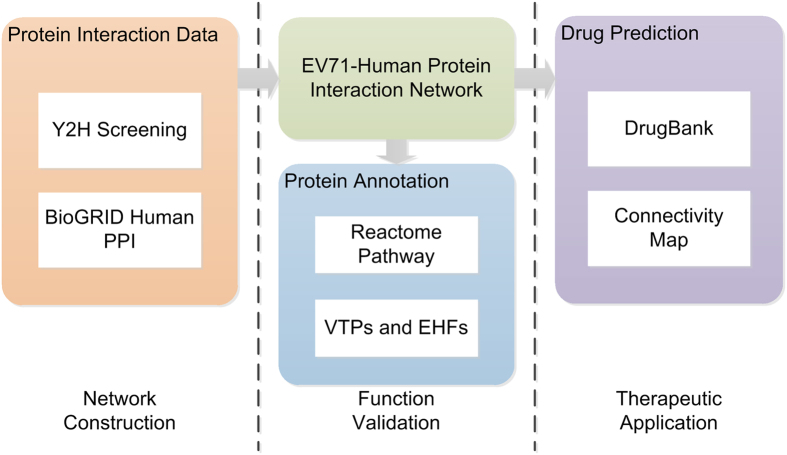
The flow chart of this study. The EV71-Human Protein Interaction Network was constructed by integrate data from public database and Y2H Screening, and used for functional annotation and drug prediction. The white blocks represent data resources.

**Table 1:** Enrichment analysis of antiviral drug candidates identified by the target match strategy.

Enrichment Term Description	Number of Candidates	Number in DrugBank	Enrichment *P* Value
Approved	264	1560	4.98 × 10^−37^
N06 (Psychoanaleptics)	37	51	3.43 × 10^−29^
D07 (Corticosteroids, dermatological preparations)	22	22	1.94 × 10^−24^
C07 (Beta-blocking agents)	19	21	6.20 × 10^−19^
Anti-inflammatory agents	19	24	9.82 × 10^−17^
Sympathomimetics	20	27	1.44 × 10^−16^
R03 (Drugs for obstructive airway diseases)	24	40	2.21 × 10^−16^
Glucocorticoids	14	14	8.91 × 10^−16^
Adrenergic uptake inhibitors	15	17	8.57 × 10^−15^
Serotonin uptake inhibitors	15	17	8.57 × 10^−15^
Corticosteroids	13	13	1.07 × 10^−14^
Anti-arrhythmia agents	26	54	1.65 × 10^−14^
Adrenergic beta-2 receptor agonists	11	11	1.54 × 10^−12^
Central nervous system stimulants	12	13	1.54 × 10^−12^
Antihypertensive agents	27	72	8.40 × 10^−12^
Dopamine uptake inhibitors	10	10	1.84 × 10^−11^
Antidepressive agents	16	28	7.06 × 10^−11^
