# Supplementary material for: Human enterovirus 71 protein interaction network prompts antiviral drug repositioning
Source: Sci Rep. 2017 Feb 21;7:43143. doi: 10.1038/srep43143 (PMC5318855; doi:10.1038/srep43143)
Supplement: Supplementary Information [file srep43143-s1.pdf]

Supplementary Information for Human enterovirus 71 protein interaction network prompts antiviral drug repositioning

Lu Han[1,2*], Kang Li[1*], Chaozhi Jin[3*], Jian Wang[3*], Qingjun Li[1], Qiling Zhang[1], Qiyue Cheng[1], Jing Yang[1], Xiaochen Bo[1], Shengqi Wang[1]

[1]Department of Biotechnology, Beijing Institute of Radiation Medicine, Beijing 100850, China;

[2]Department of Traditional Chinese Medicine and Neuroimmunopharmacology, Beijing Institute of Pharmacology and Toxicology, Beijing 100850, China;

[3]State Key Laboratory of Proteomics, Beijing Proteome Research Center, National Center for Protein Sciences (Beijing), Beijing Institute of Radiation Medicine, Beijing 102206, China.

Corresponding authors. Shengqi Wang, E-mail: sqwang@bmi.ac.cn; Xiaochen Bo, E-mail: boxc@bmi.ac.cn; Jing Yang, yangjing@bmi.ac.cn. Tel: 86-10-66931422. Address: Beijing Institute of Radiation Medicine, No.27 Taiping Road, Haidian District, Beijing, 100850, China.

[*]These authors contributed equally to this work.

Supplementary Table S1. The main classification of EIPs interacted with each viral protein.

Supplementary Table S2. The reactome pathway enrichment analysis of EIPs.

Supplementary Table S3. EAP enriched reactome pathways.

Supplementary Table S4. 2B protein interacted proteins and their neighbors enriched reactome pathways.

Supplementary Table S5. 2C protein interacted proteins and their neighbors enriched reactome pathways.

Supplementary Table S6. 3A protein interacted proteins and their neighbors enriched reactome pathways.

Supplementary Table S7. 3C protein interacted proteins and their neighbors enriched reactome pathways.

Supplementary Table S8. EV71 Nucleocapsid protein interacted proteins and their neighbors enriched reactome pathways.

Supplementary Table S9. The EIPs reported to interact with other virus proteins. Supplementary Table S10. The EIPs whose knock down have been reported to be effective in infection inhibition of other viruses.

Supplementary Table S11. Candidate drugs for anti-EV71 therapy predicted by drug target matching strategy.

Supplementary Table S12. The drug character enrichment analysis of drug-target predicted candidate drugs.

Supplementary Table S13. The drug-target action mode enrichment analysis of drug-target predicted candidate drugs.

Supplementary Table S14. Candidate drugs predicted by connectivity map.

(Supplementary Tables S3-14 were provided as separate Excel documents.)

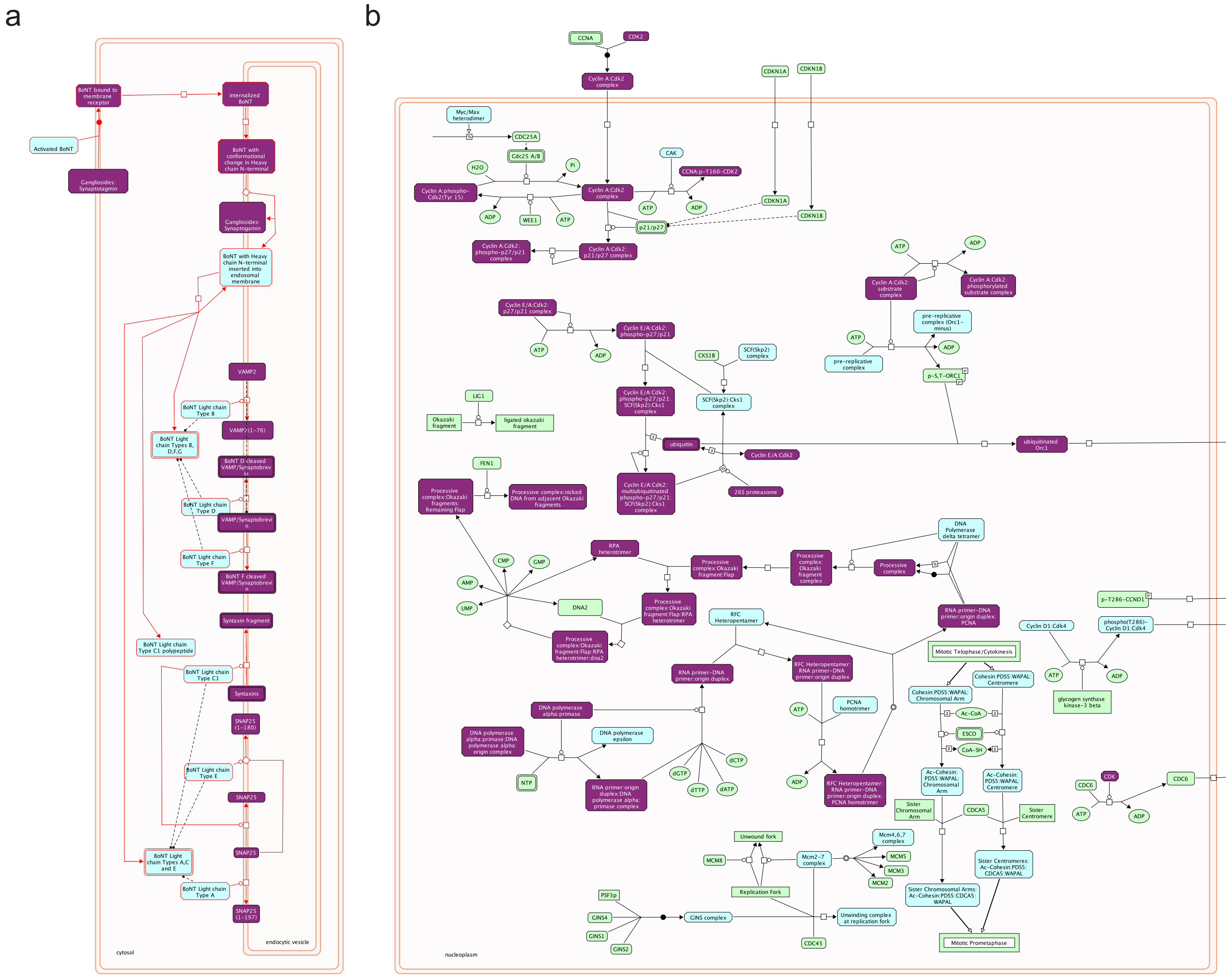

Supplementary Figure S1. EIPs and their integrated proteins enriched in the Reactome pathway. a) Botulinum neurotoxicity, 2 (STX4 and STX1A) out of the 19 proteins in Botulinum neurotoxicity were EIPs and 8 were EIP-interacting proteins; b) S phase, 33 out of 123 proteins in S phase were EIP-interacting proteins.

Supplementary Table S1. The main classification of EIPs interacted with each viral protein.

	Membrane proteins	Endoplasmic reticulum	Host-virus interaction	Glycoprotei n	Total
Nucleocapsid (VP1,VP2,VP3)	2	1	0	3	8
2B	**8**	**5**	2	1	11
2C	**3**	**3**	2	0	4
3A	**10**	3	**3**	3	10
3C	1	0	0	**2**	3
Total	18	8	6	8	29

Supplementary Table S2. The Reactome pathway enrichment analysis of EIPs.

Reactome Pathway	Number of Proteins in Pathway	Number of Proteins in EIPs	Enrich *p* value	FDR	Hit Genes
Botulinum neurotoxicity	19	2	6.0E-04	1.1E-01	STX4,STX1A
Molecules associated with elastic fibres	30	2	1.6E-03	1.6E-01	EFEMP1,FBLN5
Iron uptake and transport	36	2	2.3E-03	1.8E-01	ATP6V0C,HMOX2
Elastic fibre formation	41	2	2.9E-03	1.8E-01	EFEMP1,FBLN5
Proteolytic cleavage of SNARE complex proteins	17	2	5.0E-04	1.9E-01	STX4,STX1A
Serine biosynthesis	3	1	6.0E-03	2.4E-01	PHGDH
Glycerophospholipid biosynthesis	55	2	5.2E-03	2.5E-01	PCYT2,AGPAT1
Heme degradation	4	1	7.9E-03	2.9E-01	HMOX2
Disinhibition of SNARE formation	5	1	9.9E-03	3.6E-01	STX4
